# Social jetlag impairs balance control

**DOI:** 10.1038/s41598-018-27730-5

**Published:** 2018-06-20

**Authors:** Guilherme Silva Umemura, João Pedro Pinho, Bruno da Silva Brandão Gonçalves, Fabianne Furtado, Arturo Forner-Cordero

**Affiliations:** 1 Biomechatronics Laboratory, Department of Mechatronics. Escola Politécnica, University of São Paulo, São Paulo, Brazil; 2 Department of Psychiatry, Federal University of São Paulo, São Paulo, Brazil; 3 Federal Institute of Education, Science and Technology of Southeast of Minas Gerais, Barbacena, Brazil

## Abstract

We assessed the impact of a common sleep disturbance, the social jetlag, on postural control during a period involving workdays and free days. The sleep habits of 30 healthy subjects were registered with a wrist actimeter for nine days (starting on Friday) and they participated in a set of four postural control tests carried out on Friday and on Monday. In addition, the subjects filled questionnaires about their sleep conditions and preferences. Actimetry measurements were used to calculate the Mid Sleep Phase (MSP). The difference between the MSP values on the workdays and free days measures the social jetlag. There were significant differences in sleep variables between workdays and free days. Postural control performance improved on Monday, after free sleep over the weekend, when compared with the tests performed on Friday. It seems that social jetlag affects brain areas involved in the control of posture, such as thalamus and the prefrontal cortex as well as the cerebellum, resulting in a worse performance in postural control. The performance improvement in the posture tests after the free days could be attributed to a lower sleep debt.

## Introduction

Chronic sleep restriction is a common disturbance in which people sleep less than needed to rest properly and this has an impact on their cognitive and motor performance. This sleep disturbance affects a large portion of the population and is related to contemporary daily habits, such as delayed sleep and early wake up due to different reasons, such as work responsibilities or artificial light, resulting in a significant reduction in the quality of life^1^. If there is a conflict between the biological or chronotype driven sleeping time preferences and the times dictated by social obligations, this is named social jetlag and it can generate a sleep debt or a chronic sleep deprivation^2–4^. Chronic sleep deprivation is an important multifactorial problem that depends on sleep quantity, quality and architecture which are also related to the circadian rhythm and cycle regularity. It is associated with several health and brain function problems, loss of performance in daily tasks and in learning processes^5^. Moreover, social jetlag influences depression, smoking and metabolic disorders related to diabetes, obesity and increased cardiovascular risk, and is related to cognitive deficits due to disturbances in the prefrontal cortex function caused by sleep deprivation^2–4,6^.

In order to explain how the social jetlag affects the sleep, it is necessary to understand how the timing system of the sleep-wake cycle works. To explain this phenomenon, a process involving circadian and homeostatic regulation of sleep was proposed^7,8^. The homeostatic regulation process (S process) in which the pressure of the sleep increases exponentially from the beginning of the wake until the beginning of the sleep, recovering from this moment, it acts along with a circadian component (process C) in which there are phases of the day with higher or lower propensity to sleep.

This model also predicts that if there is no complete recovery of the accumulation of sleep pressure during the rest phase (i.e., sleeping less than necessary or with a poor sleep quality), the individual cannot recover all the sleep pressure accumulated during the previous wakefulness. This generates a deficit in homeostatic control of the sleep-wake cycle, and consequently causes problems related to lack of sleep, such as drowsiness, decreased attention, among other cognitive disorders.

It is commonly considered that weekend sleep is used to compensate for chronic low sleep quality^4^. It is also known that, after a long time awake, the postural stability performance is worsened, showing larger variance in the excursion of the body center of pressure^9^, and it has been used to evaluate sleepiness^10,11^. Moreover, in a recent work, a group of volunteers with worse sleep quality showed a lower performance in postural control tests with eyes closed, suggesting that the lack of vision affects postural control in subjects with chronic sleep inefficiency^12^.

There are some recommendations about the minimum sleep time that for adults is between seven and nine hours^13^. These recommendations are based on expert panels and systematic literature reviews^14^. Nevertheless, as those authors pointed out, there is a lack of objective measurements of the sleep conditions, involving not only time but also quality or architecture, particularly for large cohort studies^14^. Therefore, objective sleep measurements are necessary^15^. In this respect, the performance evaluation of certain tasks, either motor or cognitive, that can be related to the chronic sleep condition of the subject, would be an interesting procedure to complement sleep evaluation tools.

The social jetlag is a phenomenon that causes a reduction in the sleep time^4^. It has been shown that sleep restriction raises the prevalence of several diseases^3,16^ and causes a reduction in the neurocognitive function^5^. The postural control depends of the optimal functioning of the central neural system (CNS) in areas like pre frontal cortex and cerebellum^17^. These areas of the CNS are the two of the most affected by sleep disturbances^18,19^ and causes a deficit in the real time control of the posture, which may also disturb the control of the gait. In this study, we examined the influence of sleep disturbances on postural control with actigraphy, instead of questionnaires, as in other social jet lag studies^6^. Sleep was assessed with actimetry, during a period including working days and free days; afterward, the subjects performed a set of postural control tests and filled sleep questionnaires. Therefore, our goal was to compare the effects of sleep weekly routines on postural control with a group of healthy adults.

## Methods

### Participants

Thirty healthy (body mass index of 22.21 ± 3.72 kg/m^2^) undergraduate students (21.3 ± 2.2 years of age) of both genders (35% male) participated in the study. This research was performed in accordance with relevant guidelines/regulations and each subject gave a written informed consent to participate in the study that was approved by the Ethics Committee of the Federal Institute of Education, Science, and Technology of Southeast of Minas Gerais and registered in the official Federal database. Exclusion criteria included pregnancy, diagnoses of psychiatric or sleep disorders, alcohol or caffeine dependence and recent transmeridian travel. According to the score obtained by the International Physical Activity Questionnaire, all the subjects were classified between the descriptors irregularly active and active. The circadian preference was assessed using the Horne and Ostberg Morningness/Eveningness Questionnaire. While cognitive function and mood were not evaluated, the interviewer that applied the questionnaires did not report any irregular behavior of the participants regarding cognitive functions or mood.

### Questionnaires

The Pittsburgh Sleep Quality Index Questionnaire or PSQI^20^ and the Epworth Sleep Scale or ESS^21^ are two questionnaires to evaluate, respectively, sleep quality and daytime sleepiness. They were filled on the last day of actimetry recordings, before the last postural control tests. The PSQI is composed of nineteen individual items that generate seven “component” scores: subjective sleep quality, sleep latency, sleep duration, habitual sleep efficiency, sleep disturbances, use of sleeping medication, and daytime dysfunction. The score of the answers is based on a 0 to 3 scale. A global sum of the components equal or greater than “5” indicates sleep problems.

A local adapted version of the Morningness–Eveningness Questionnaire (MEQ)^22^, also known as HO questionnaire, developed by Horne an Östberg, was used^23^. It measures whether a person’s circadian rhythm produces peak alertness in the morning, in the evening, or in between. The respondent is asked to indicate when, he/she would prefer to wake up or start sleep, according to time availability. The scores are added and converted into a five-point scale: 16 to 30 score “definitely evening type”; 31 to 41 score “moderately evening type”, 42 to 58 score “neither type; “59 to 69 score “moderately morning type; and 70 to 86 score “definitely morning type”.

Finally, the ESS is a questionnaire that measures a person’s general level of daytime sleepiness. The respondents must rate, on a 4-point scale (0–3), their usual chances of dozing off or falling asleep. The higher the score, the higher the person’s level of daytime sleepiness. Using a total cut-off score >10, it is possible to identify individuals with excessive daytime sleepiness. Scores >16 indicate severe sleepiness. There were two ESS scores for each subject, one for the weekdays and the other for the weekends.

### Sleep-wake monitoring

The participants wore an actimeter (ActTrust®, Condor Instruments Ltda, SP, Brazil) continuously for nine days before filling the questionnaires. They were instructed to follow their normal daily routines. The devices were configured to register and process the activity with Proportional Integral Mode (PIM) algorithm (1-minute epoch). The sleep variables were calculated using the software ActStudio (Condor Instruments Ltda., SP, Brazil) and the data were grouped as workdays (Monday to Friday) and free days (weekends). The sleep parameters obtained were: nocturnal activity L5 (sum of activity during the 5 hours of less movements), daily activity M10 (sum of activity during the 5 hours of less movements), sleep onset (SON), sleep offset (SOFF) and total sleep time (TST).

### Social jet lag

The Mid-sleep phase (MSP) is the best phase anchor point for melatonin onset, a robust circadian phase marker^24^. Therefore, the MSP difference between workdays and free days is a measure of social jetlag^4^. The presence of significant MSP differences between work and free days is one of the more frequently used indicators of social jetlag^4^. The MSP was calculated using the actimeter variables total sleep time (TST) and sleep onset (SON), separately for work and free days, according to Equation 1:

1 $${\rm{MSP}}={0.5}^{\ast }{\rm{TST}}+{\rm{SON}}$$

### Postural control performance

Postural control was assessed with the Biodex Balance System® (BBS, Biodex Inc Shirley, NY USA). The BSS consists of a circular balance platform that provides up to 20° of surface tilt in a 360° range of motion and can move in the anterior-posterior and medial-lateral axes simultaneously. The BBS software (Biodex, Version 1.08, Biodex, Inc., Shirley, NY USA) allows the control and measurement of the platform data. It is possible to include a foam pad under the feet of the participant to perturb sensory information. The stability index (SI), provided by BBS, is the score based on the variance of the platform displacement (anteroposterior stability index – APSI, medio-lateral stability index – MLSI, and overall stability index (OSI). Higher values of SI indicate worse postural control as this displacement is related to the center of pressure (COP) excursions. The sway index, provided in the static platform tests, is the standard deviation of the SI.

The tests were conducted at the same time of the day in both assessments to minimize the influence from the circadian rhythm. The subjects were asked to stand barefoot on the platform with the feet slightly apart and the arms hanging. A grid printed on the platform was used to maintain the feet in the same position in each experimental condition and in different days. Familiarization trials were allowed to ensure that the tasks were properly understood and learnt before the recordings.

Four tests were used to assess the influence of sleep debt and social jet lag on several factors of postural control such as the reduction of kinesthetic and visual information, in the ability to react to changes in the base of support (platform tilt) or in the ability to actively control the posture. The BBS system allows to change the platform resistance to tilting as the COP moves on the platform, that is referred as platform rigidity. If the resistance is higher, it is a more rigid platform condition that offers a firm support. As the resistance is lower, the platform tilts becoming a more challenging condition for posture control.

A static postural test was performed with plantar pressure sensory deprivation (a foam under the platform) performed with (eyes closed - SPT EC) or without visual information deprivation (eyes open - SPT EO); a fixed instability postural test with eyes open (FPT EO) or closed (FPT EC), with no sensory deprivation; a variable instability postural test with eyes open (VPT EO) or closed (VPT EC), with no sensory deprivation; and a dynamic postural test with high (DPT H) or low (DPT L) instability, with no sensory deprivation.

Static postural test (SPT): with a soft foam plate on top of the platform, the participants were asked to step on the foam and assume a quiet stance for 30 seconds.

Fixed instability postural test (FPT): for 30 seconds the platform offered a fixed instability condition (platform tilting) and the participants were asked to maintain their center of pressure (COP) still.

Variable instability postural test (VPT): for 30 seconds the platform offered a progressively decreasing stability (from high to low platform rigidity conditions) while the participants were asked to maintain their COP still.

Dynamic postural test (DPT): the participant was asked to move a cursor on that displays the COP position in real-time on a screen, to reach several cardinal and intercardinal points, that appeared randomly, as quickly and as straightly as possible.

All the postural test conditions were performed twice, with a two-minute rest between tests. The mean sway index in the first three conditions was used as the measure of postural control, i.e., higher values mean worse postural performance. In the last condition, a scoring percentage reflects the directional accuracy of the movement, meaning that lower values represent worse postural performance.

### Statistical analysis

The statistical procedures were carried out using SigmaStat 3.5 (Systat Inc., Germany). Initially, the raw data was visually inspected to detect possible outliers. Then, Leven and Shapiro-Wilk tests were conducted to attest homoscedasticity and normality distribution, respectively. To compare the differences between days, a two-tailed paired T-test with Bonferroni adjustment and with a significance level set at 0.05 was conducted. The magnitude of the differences was indicated by the Dunlap effect size and their respective 95% confidence intervals^25^.

## Results

The chronotype classification in MEQ (48.83 ± 9.48 score points 9:45 ± 1:37 hours of desired hours of sleep) and the assessment of the sleep quality index (6.87 ± 3.22 score points in the PSQI), yielded normal values for this population. Afterwards, the sleep parameters were compared between workdays and workdays. The mean and standard deviation of the sleep variables are shown in Table 1.

**Table 1:** Mean and standard deviation nocturnal activity (L5), daily activity M10, sleep onset (SON) and offset (SOFF), total sleep time (TST), Epworth sleepiness scale questionnaire (ESS) and mid sleep phase (MSP) on workdays and free days.

Variable	Workdays	Free Days	t	P
L5	125.5 (55.7)	133.3 (71.3)	−0.515	0.611
M10	5511.7 (1906.1)	5230.9 (1457.8)	1.088	0.287
SON (hh:mm)	23:46 (01:16)	00:50 (01:37)	−2.807	0.010
SOFF (hh:mm)	07:38 (01:16)	09:18 (01:43)	−5.574	<0.001
TST (hours)	7.69 (1.00)	8.48 (1.03)	−3.306	0.003
ESS (score)	8.60 (3.96)	9.00 (3.81)	−1.418	0.167
MSP (hh:mm)	03:42 (01:11)	05:04 (01:35)	4.382	<0.001

T-values and p-values from the paired t-test are also shown.

The mean and standard deviation of the postural control variables are shown in Table 2.

**Table 2:** Mean and standard deviation of the eight postural control conditions stability index (SI) and accuracy (%) on Friday and on Monday.

Variable	Friday	Monday	t	P
SPT EO (SI)	1.06 (0.31)	1.13 (0.37)	−1.542	0.134
SPT EC (SI)	2.52 (0.65)	2.58 (0.57)	−0.688	0.497
FPT EO (SI)	1.54 (0.67)	1.28 (0.41)	2.622	0.015
FPT EC (SI)	5.44 (2.20)	5.24 (2.07)	1.423	0.167
VPT EO (SI)	1.48 (0.53)	1.36 (0.63)	1.005	0.323
VPT EC (SI)	3.88 (1.73)	3.36 (1.42)	2.985	0.006
DPT L (%)	32.05 (7.51)	37.67 (7.86)	−5.034	<0.001
DPT H (%)	33.17 (8.47)	36.97 (8.91)	−3.533	0.001

T-values and p-values from the paired t-test are also shown.

SPT-static postural test; FPT-Fixed instability postural test; VPT-Variable instability postural test; DPT-Dynamic postural test; EO-eyes open; EC-eyes closed; L-low instability; H-high instability.

**Figure 1:**
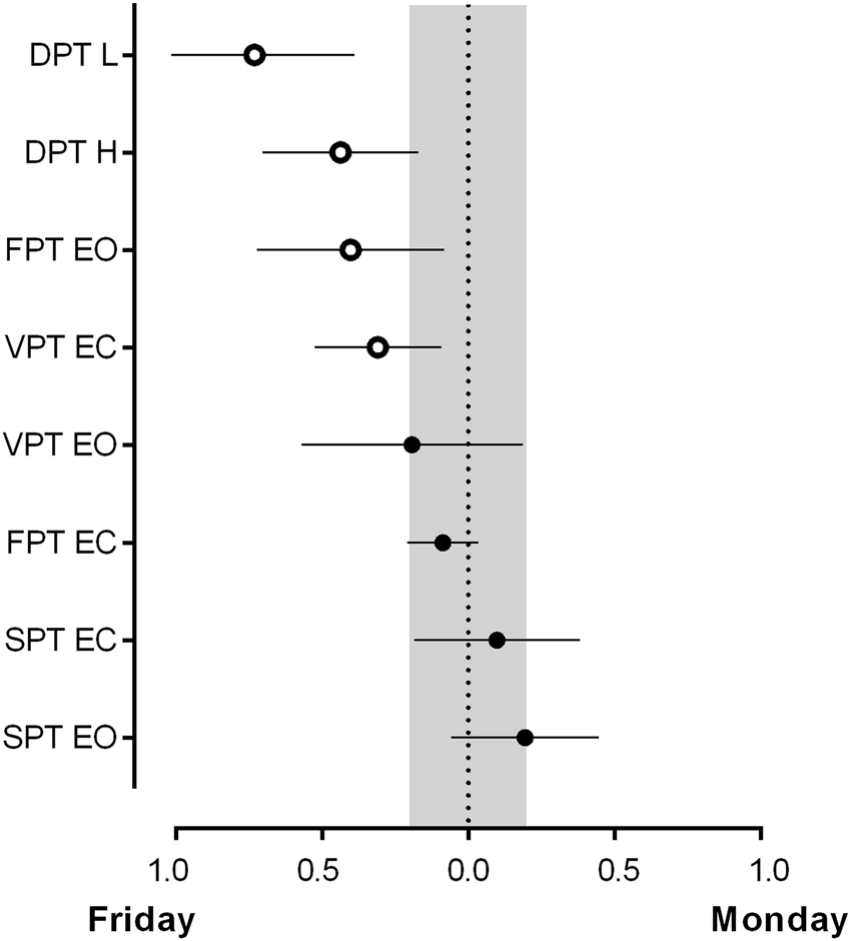
Dunlap effect size and 95% confidence intervals of the differences between workdays and free days. A blank mark denotes a significant difference (p < 0.05) between days and a solid mark the absence of significant differences. The shaded area specifies the interval at which the effect size of the difference between days is trivial (−0.2 < *d* < 0.2). SPT-static postural test; FPT-Fixed instability postural test; VPT-Variable instability postural test; DPT-Dynamic postural test; EO-eyes open; EC-eyes closed; L-low instability; H-high instability.

The significant sleep differences in several parameters between the working days and the weekends indicate the possibility of “social jetlag” in this population. The posture variables also showed significant differences. The magnitude of the differences in the postural variables between Monday and Friday is presented in Fig. 1. Posture control performance was consistently better on Mondays than on Fridays.

## Discussion

The control of posture is well known to be affected by acute sleep deprivation^11^, however, the effects of chronic sleep restriction due to sleep debt and social jet lag have received less attention^12^. Our hypothesis is that sleep disruption due to the weekly routine affects postural performance. Hence, after sleep recovery during the weekend, individuals are expected to perform better in postural control tests. We assessed a group of volunteers that used an actimeter for weekends and weekdays before performing a posture control test. From the data collected by actimetry, it was possible to quantify the total sleep time of weekdays and weekends and use this information to compare postural control performance.

Interestingly, the total sleep time during the week was about almost one hour less when compared with the total sleep time during weekends. However, the total sleep times were, on average, above 7:40 h during the week and more than 8 hours at the weekends, which cannot be regarded as sleep restriction according to the most recent recommendations found in the literature^5^. However, when asked in the Horne and Ostberg Questionnaire how many hours of sleep were desired (9:45 h), the subjects sleep at least two hours less during weekdays and at least one hour less during weekends than preferred. These findings show that the individuals in this study suffered from a shortening of hours of sleep during the week that may not be fully restored at weekends. While sleeping, it is assumed that micro-arousals, as well as other events, would result in more movement that can be recorded by the actimeter. There are actimetry parameters, such as L5, that measure the movement during sleep and, thus give an index of sleep quality^12,26^.

While L5 measures the activity in the 5 hours with less motion, M10 measures the activity during the 10 hours of more motion. However, L5 and M10 showed no significant differences between work and freedays, so, activity differences or sleep quality are unlikely to account for the postural control differences found in this work.

Moreover, the reported sleepiness from the ESS questionnaire is very similar on Friday and on Monday and presents the results expected for a group without disturbances or sleep-related diseases.

Remarkably, the subtle sleep differences between weekdays and weekends seem to affect postural performance. Based on our findings, we can state that the same group of people have different postural control performance in working and non-working days. We significantly reduced the probability that these results are due to chance (statistically significant differences) and, since all major factors were controlled, we can attribute it to the measured sleep debt.

In general, improvement in the performance can be observed on Monday, after unrestricted sleep over the weekend, when compared with the tests performed on Friday, in which there is a restriction in the total sleep time, thus generating a sleep debt.

Postural control relies on the integration of visual, somatosensory and vestibular information^17^. The latter, which provides information about the three-dimensional head rotations and translation^26^, has pathways that involve the thalamus and the vestibulothalamic projections. These pathways have multiple presentations of the vestibular information in the cerebral cortex^17^.

The major function of the thalamus is to relay motor and sensory signals to the cerebral cortex, however, it is also involved in signal processing within complex cortico-thalamo-cortical loops having a fundamental role in the thalamus arousal, allowing a modulation of the information transmitted to the cortex^27^. Thus, a lower excitability of the thalamus may indicate a decrease in the transmission of sensory information to other parts of the brain. Several areas of the central nervous system are important for postural control, and two of the most important areas are the prefrontal cortex and cerebellum^17,21^. The cognitive postural control depends on cognition of self-body information together with spatial localization. The cerebellum regulates the cognitive and automatic processes of postural control by acting on the cerebral cortex^17^.

Chronic sleep restriction over 14 days resulted in cognitive performance deficits equivalent to up to 2 nights of total sleep deprivation^28^. In other words, even moderate sleep restriction can disturb the cognitive functions^29^. In the condition with the highest cognitive demand (DPT), it was observed the largest divergence in postural control performance between the tests carried out on Friday and on Monday. Postural performance on Monday is significantly higher than on Friday. These results are consistent with the hypothesis that sleep restriction affects the pre-frontal cortex, which is primarily responsible for aspects of higher cognitive demand.

Standing on a force platform with eyes closed and a foam under the feet alters plantar and ankle proprioception. In the tasks performed without visual information and with reduced plantar and ankle proprioception, the control of posture must rely on vestibular and proprioceptive inputs to estimate the configuration of the body with respect to the environment^30,31^. The sensory perturbations require higher processing demands on certain cortical and cerebellar areas; however, no significant differences between Monday and Friday were found, independently of the visual condition (eyes open or closed).

Connections of the prefrontal cortex with regions of the cerebellum are responsible for the real-time control of movement execution; different areas of the cerebellum are very important for locomotion and postural adjustments, as well as complex movements associated with auditory and visual stimuli, such as reaching with a hand at a given point along with a visual stimulus^32–34^. This circuitry plays a crucial role in performing voluntary movements, especially when these movements require precision and fine motor skills, as well as in sequences of movements involving many joints^17^.

Studies analyzing functional metabolism and brain neurophysiology demonstrate that neural systems involved in executive functions are affected by the sleep loss, even with partial sleep restriction^19^. One of these conditions of sleep disruption is the social jetlag, one of the most common disturbances in the daily routine^35^. Sleep problems affect cognitive function and may deteriorate the response time, the process of task acquisition and the task execution performance in a short period of time, as well as an increase in the effort in the implementation of the tasks in an efficient way^19,36^.

Furthermore, it is possible that required sleep time and the current lower limit in the number of hours sleeping for young adults^15^, that is set to seven^13^, could be underestimated and may not allow the subject to achieve the best motor performance. This would explain why young healthy adults with a small unnoticed chronic sleep restriction have lower postural control performance. Moreover, postural control results could be used as a complementary objective measurement of sleep restriction. Future research including posture control measurements in the assessment of sleep for long periods with actimetry and questionnaires is guaranteed to determine the relationship between sleep restriction and posture and the possible implications in populations with stability problems, such as the elderly or patients suffering from vestibular pathologies.
